# A novel immune-isolation method for direct quantification of triglycerides associated with lipoprotein(a)

**DOI:** 10.1016/j.jlr.2026.100996

**Published:** 2026-02-10

**Authors:** Lizhu Lin, Fei Su, Calvin Yeang, Sotirios Tsimikas

**Affiliations:** Vascular Medicine Program, Division of Cardiovascular Medicine, Univeristy of California San Diego, La Jolla, CA, USA

**Keywords:** cholesterol, lipoprotein(a), triglycerides

## Abstract

Lipoprotein (a) [Lp(a)] is viewed as a cholesterol-rich, LDL-like particle, yet potential heterogeneity in its lipid composition is not well understood. We developed and validated a novel immune-isolation assay to directly quantify triglycerides (TGs) associated with Lp(a) [Lp(a)-TGs]. Lp(a) was selectively isolated from plasma using magnetic beads conjugated with monoclonal antibody LPA4 targeting apolipoprotein(a), followed by enzymatic quantification of TGs. Assay specificity was ensured using washing buffers to prevent nonspecific lipoprotein interactions. Spike-in experiments with purified VLDL/intermediate density lipoprotein lacking Lp(a) demonstrated no measurable interference. Lp(a)-cholesterol [Lp(a)-C] was measured using an established immune-isolation method. The ratio of Lp(a)-TG to Lp(a)-C was calculated to distinguish TG-enriched Lp(a) particles from the typical cholesterol-rich, LDL-like phenotype. Lp(a)-TG, Lp(a)-C, Lp(a) molar concentration, and estimated compositional ratios were quantified in 36 normotriglyceridemic individuals and 114 individuals with moderate hypertriglyceridemia (150–500 mg/dl). In normotriglyceridemic individuals, mean (SD) TGs were 98.4 (31.9) mg/dl, Lp(a)-TG 1.42 (2.83) mg/dl, Lp(a)-C 4.03 (4.01) mg/dl, and the Lp(a)-TG/Lp(a)-C ratio was 0.59 (1.27). Lp(a)-TG and Lp(a)-C accounted for mean (SD) 1.22% (0.10) of total plasma TGs and 2.62% (2.01) of total plasma cholesterol. In individuals with hypertriglyceridemia, mean (SD) TGs were 284 (85) mg/dl, Lp(a)-TG 53.7 (25.3) mg/dl, Lp(a)-C 14.4 (6.9) mg/dl, and the Lp(a)-TG/Lp(a)-C ratio was 3.99 (1.20). Lp(a)-TG and Lp(a)-C accounted for mean (SD) 19.9% (6.53) of total plasma TGs and 9.68% (4.41) of total plasma cholesterol. This immune-isolation assay is the first validated, high-throughput method for direct quantification of Lp(a)-TG. This study demonstrates that Lp(a) lipid composition is variable and enriched in triglycerides and cholesterol in hypertriglyceridemic states. It provides a platform for future mechanistic, epidemiologic, and pharmacologic studies of Lp(a)-triglyceride interactions. This immune-isolation assay is the first validated, high-throughput method for direct quantitation of Lp(a)-TG.

Lipoprotein(a) [Lp(a)] is a genetically determined lipoprotein particle composed of apo(a) covalently bound to apolipoprotein B-100, conferring both LDL-like lipid characteristics and unique structural heterogeneity (1). The size and composition of Lp(a) particles vary widely due to genetically determined kringle IV_2_ repeat number, extensive glycosylation of apo(a) adding up to 30% of its mass, and variable lipid content. The lipoprotein component contains apoB-100, cholesteryl esters, free cholesterol, phospholipids, and triglycerides (TGs). Lp(a) is referred to as an “LDL-like” particle as it generally has the density and size most typical of LDL particles in normolipidemic individuals (2). Using ultracentrifugation techniques, in normolipidemic individuals the majority (>90–95%) of Lp(a) is found in density regions 1.050–1.120 g/ml that overlap with higher density LDL particles and the entire range of HDL particle densities (3). Furthermore, in normolipidemic individuals the TG content of purified Lp(a) is low, ranging from 2% to 8% of the total lipid mass (4, 5).

However, despite extensive investigation of Lp(a) as a causal risk factor for atherosclerotic cardiovascular disease, its relationship with TG metabolism remains incompletely understood. Epidemiologic studies consistently demonstrate a modest inverse correlation (r = −0.10–0.20) between plasma TGs and Lp(a) immunoassay concentrations, suggesting metabolic interplay between triglyceride-rich lipoproteins (TRLs) and apo(a)-associated particles (6, 7, 8, 9). Earlier ultracentrifugation and electrophoretic studies suggested the presence of apo(a) immunoreactivity within TRL density fractions, but these approaches were limited by labor-intensive fractionation, small sample sizes, pooled plasma, and the inability to distinguish intrinsic lipid content of Lp(a) from comigrating remnant lipoproteins (10, 11, 12, 13, 14, 15, 16, 17, 18). As a result, the magnitude of TG enrichment on Lp(a) particles and its relationship to plasma TG burden remain poorly defined.

To address this gap, we developed and validated a novel immune-isolation assay that directly quantifies TGs associated with apo(a)-containing particles [Lp(a)-TG]. Using a monoclonal antibody (mAb)-based isolation strategy coupled with enzymatic lipid quantification, this method enables sensitive, interference-resistant measurement of Lp(a)-TG. We applied this assay across normotriglyceridemic and hypertriglyceridemic individuals to define the distribution and metabolic correlates of Lp(a)-TG.

## Materials and Methods

### Study populations

Two cohorts were studied. A normotriglyceridemic control group consisted of 36 consecutive individuals undergoing clinically indicated coronary angiography with fasting plasma TG levels <175 mg/dl at the University of California Medical Center. Plasma samples were obtained in the fasting state from the arterial sheath immediately prior to angiography. All participants provided written informed consent, and the study was approved by the University of California San Diego Human Subjects Protection Program.

The moderate hypertriglyceridemic cohort (19) was derived from a randomized, placebo-controlled phase 2 study of apoC-III inhibition and consisted of 114 adults with fasting TG levels between 200 and 500 mg/dl and either established atherosclerotic cardiovascular disease or high cardiovascular risk. The mean age was approximately 65 years, 75% were male, and 75% had coronary artery disease and multiple cardiometabolic risk factors, including hypertension (89%), type 2 diabetes (68%), and prior hypercholesterolemia (73%). Most participants were receiving background lipid-lowering therapy, including statins (84%) and fibrates and/or omega-3 fatty acids (40%). Baseline median fasting TG levels were 262 mg/dl (interquartile range [IQR] 222–329), with elevated apoC-III and VLDL-cholesterol (VLDL-C) concentrations, modestly reduced HDL-C, and LDL-C levels generally within guideline-recommended ranges.

The protocol was approved by the institutional review board at each participating center and by an ethics committee (Quorum Review IRB), and the trial was performed in accordance with the principles of the Declaration of Helsinki and current Good Clinical Practice guidelines. All patients provided written informed consent before enrollment.

### Lipid variables

Blood samples were collected into EDTA-containing tubes following an overnight fast and processed and stored at −70°C until analysis. Samples underwent no thawing cycles prior to assay measurements. Lipid parameters in the normocholesterolemic cohort were performed at University of California San Diego (UCSD) Clinical laboratory with standard commercial reagents. LDL-C was estimated using the Martin-Hopkins equation. In the moderate hypertriglyceridemia cohort, lipid parameters were measured at apol Reference laboratories with standard commercial reagents. LDL-C was measured for following ultracentrifugation with a direct LDL method and VLDL-C was estimated by subtracting total cholesterol from LDL-C and HDL-C.

### Lp(a) assays

For the UCSD cohort, Lp(a) was measured in nmol/L by an in-house isoform-independent method as previously described (20, 21). In the moderate hypercholesterolemia cohort, Lp(a) was measured in nmol/L using the Roche Tina-quant Gen. 2 kit on a Roche c502 analyzer. Each of the five standards used to calibrate the assay is formed by plasma pools with apo(a) isoforms ranging from large to small and Lp(a) concentrations from low to high. In both methods, the target values to each standard were independently assigned against the World Health Organization/nternational Federation of Clinical Chemistry SRM-2B reference material.

### Isolation of VLDL and IDL

Purified pooled VLDL and intermediate density lipoprotein (IDL) fractions were prepared from plasma obtained from a donor with very low Lp(a) concentration (12 nmol/L) to assess potential TRL interference in the immune-isolation assay. Whole blood was collected into EDTA tubes and centrifuged at room temperature at 1,300 *g* for 10 min to separate plasma. Plasma was subjected to two sequential room temperature ultracentrifugation steps at 50,000 rpm for 15 min to remove chylomicrons. The density of the chylomicron-depleted plasma was then adjusted to 1.020 g/ml using NaBr and ultracentrifuged at 75,000 rpm at 4°C for 16 h. The upper fraction containing VLDL and IDL was carefully collected and buffer-exchanged into 1× PBS using 300 kDa molecular weight cutoff centrifugal filter units by diafiltration at 2,800 *g* at 10°C.

### Assessment of interference from TG-rich lipoproteins in the Lp(a)-TG and Lp(a)-C assays

To evaluate potential assay interference from TG-rich lipoproteins lacking apo(a), purified VLDL and IDL fractions free of Lp(a) spiked into plasma samples containing either low [Lp(a) 9 nmol/L] or high [Lp(a) 520 nmol/L] Lp(a) concentrations, or into assay buffer (vehicle control). Increasing concentrations of VLDL-C/IDL-C or TGs were added prior to immune-isolation with LPA4-conjugated magnetic beads. Following immunocapture and washing, Lp(a)-cholesterol [Lp(a)-C] and Lp(a)- Lp(a)-TG were quantified enzymatically.

### Quantification of Lp(a)-TGs

**Fig. 1:**
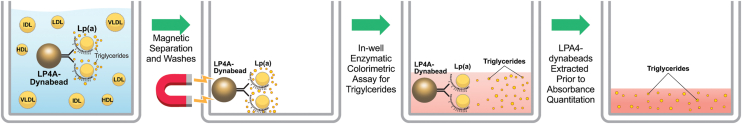
Methodology of the Lp(a)-TG assay. Schematic representation of the immune-isolation assay used to quantify triglycerides associated with lipoprotein(a) [Lp(a)-TG]. Apolipoprotein(a)-containing particles are selectively isolated from plasma using magnetic beads conjugated with the monoclonal antibody LPA4, followed by magnetic separation, and washing steps performed in washing buffer containing proline and ε-aminocaproic acid (EACA) to prevent nonspecific lipoprotein interactions. Isolated Lp(a) particles are incubated directly in-well with enzymatic triglyceride reagent, after which LPA4-conjugated beads are removed prior to absorbance measurement. Triglyceride concentrations are determined spectrophotometrically using standard curves corrected for plasma input volume. Lp(a), lipoprotein(a).

Lp(a)-TG were quantified using an immune-isolation assay based on magnetic beads conjugated with the apo(a)-specific mAb LPA4 (Fig. 1). Apo(a) was selectively captured using MyOne Epoxy magnetic beads (Life Technologies) conjugated with the murine mAb LPA4, which recognizes a conserved epitope within apo(a). LPA4 binds the 14 amino acid peptide TRNYCRNPDAEIRP present on apo(a) KIV_5_, KIV_7_, and KIV_8_, as well as the partial sequence NYCRNPDA within KIV_2_, ensuring high-affinity, isoform-independent capture of Lp(a) without cross-reactivity to apoB or plasminogen. Because this assay is designed for immune-isolation rather than quantification of apo(a) molar concentration, the high-affinity, multivalent binding of LPA4 to multiple apo(a) kringle epitopes is advantageous, ensuring efficient and specific capture of all apo(a)-associated particles across apo(a) isoform sizes.

Conjugation of LPA4 to MyOne Epoxy Dynabeads was performed as previously described, using 30 μg of antibody per milligram of beads (22). LPA4-Dynabeads (40 μl per well) in PBS were dispensed into 96-well round-bottom plates and the PBS was removed. To disrupt nonspecific interactions of lipoproteins, ε-aminocaproic acid (EACA) was added consistently to all experiments to competitively inhibit lysine-dependent kringle interactions of apo(a), and proline is added to reduce low-affinity hydrophobic and protein-protein interactions that facilitate transient association of TRLs with apo(a)-containing particles. Importantly, neither agent disrupts the covalent apo(a)–apoB linkage or particle integrity. Plasma samples were diluted in washing buffer (1% BSA, 200 mM proline (Sigma-Aldrich, St Louis, MO), 200 mM EACA [Acros Organics, Geel, Belgium] in PBS, pH 7.2) according to Lp(a) concentration: samples with Lp(a) > 200 nmol/L were diluted 1:10, whereas samples ≤200 nmol/L were diluted 1:1. Diluted samples were mixed and equilibrated by shaking prior to immunoprecipitation. Beads and the plasma were incubated under agitation (550 rpm) at room temperature to allow capture of Lp(a) particles for 45 min, followed by magnetic extraction and five sequential washes with washing buffer to remove nonspecifically bound lipoproteins and plasma components. Under these conditions, complete extraction of detectable apo(a) has been previously demonstrated in the context of Lp(a)-C measurement (22).

After the final wash, bead-bound Lp(a) particles were incubated with enzymatic TG reagent (Pointe Scientific) using a glycerol-blanked enzymatic method in a flat-bottom 96-well plate. This method measures total glycerides; however, because measurements were performed on bead-bound apo(a)-associated particles after multiple wash steps, contribution from free glycerol is expected to be negligible. TG standards were prepared by serial dilution from a single commercial calibrator and included on each assay plate. Following incubation at 37°C for 5 min, beads were magnetically removed to allow optical clearing, and TG content was quantified by spectrophotometric measurement at 500 nm with background correction at 700 nm. Samples were analyzed in duplicate, with low and high plasma sample calibrators included on each plate. Lp(a)-TG concentrations were derived from a TG standard curve (linearity r = 0.9994), corrected for plasma input volume, and demonstrated an intra-assay coefficient of variation of 3.5%. All samples were stored at −70°C and underwent no freeze–thaw cycles prior to analysis.

### Measurement of Lp(a)-C and Lp(a) molar concentration

Lp(a)-C was measured using a previously validated immune-isolation assay with enzymatic cholesterol quantification (22, 23). The assay has a sensitivity <1 mg/dl and a dynamic range extending to 747 nmol/L Lp(a). Lp(a) molar concentration (nmol/l) in the UCSD cohort was measured with an isoform-independent ELISA using mAbs LPA4 as the capture antibody and biotin-LPA-KIV9 as the detection antibody, respectively, as previously described (20, 21, 24). In the hypertriglyceridemia cohort Lp(a) in nmol/L was measured using Roche Tina-quant Gen. Two kit on a Roche c502 analyzer at Medpace Reference Laboratories.

### Derivation of Lp(a) compositional ratios: Lp(a)-TG/Lp(a)-C, Lp(a)-TG/Lp(a), and Lp(a)-C/Lp(a)

It is accepted that the TG to cholesterol ratio (in mg/dl units convention for VLDL and LDL) is ≤ 5 in individuals without hypertriglyceridemia as initially documented by Friedewald, whereas higher ratios represent the presence of TRLs (25, 26). We modified this convention to assess the ratio of Lp(a)-TG/Lp(a)-C using mg/dl units as an index of Lp(a) reflecting a larger TRL-like particle rather than the typical LDL-like particle. Furthermore, Lp(a)-TG/Lp(a) and Lp(a)-C/Lp(a) molar ratios were calculated by converting TG and cholesterol mass concentrations to molar units and normalizing to Lp(a) particle number in nmol/l, enabling assessment of lipid content per Lp(a) particle.

For the Lp(a)-TG/Lp(a)-C ratio, both variables were expressed in mg/dl, and the ratio was calculated directly as a dimensionless value. For the Lp(a)-TG/Lp(a) molar ratio, TG concentrations (mg/dl) were converted to molar units by dividing by 88.57 to obtain mmol/l, followed by multiplication by 1,000,000 to obtain nmol/l. This value was then divided by the Lp(a) molar concentration (nmol/l) to derive the molar ratio. Similarly, for the Lp(a)-C/Lp(a) molar ratio, cholesterol concentrations (mg/dl) were converted to mmol/l by dividing by 38.67 and then to nmol/l by multiplication by 1,000,000 before normalization to Lp(a) molar concentration. These ratios reflect the estimated number of TG or cholesterol molecules per Lp(a) particle.

### Statistical analyses

Continuous variables are presented as mean ± SD or median with IQR, as appropriate. Correlations between Lp(a)-TG, Lp(a)-C, Lp(a)-TG/Lp(a)-C ratio, and other lipid parameters were assessed using Spearman correlation analysis. The correlation of these variables with other lipid analytes at the primary analysis timepoint was assessed using partial Spearman correlation analysis, controlling for treatment and log-transformed baseline values. Statistical analyses were performed using SAS version 9.4 (Cary, NC).

## Results

### Assay specificity and interference testing

**Fig. 2:**
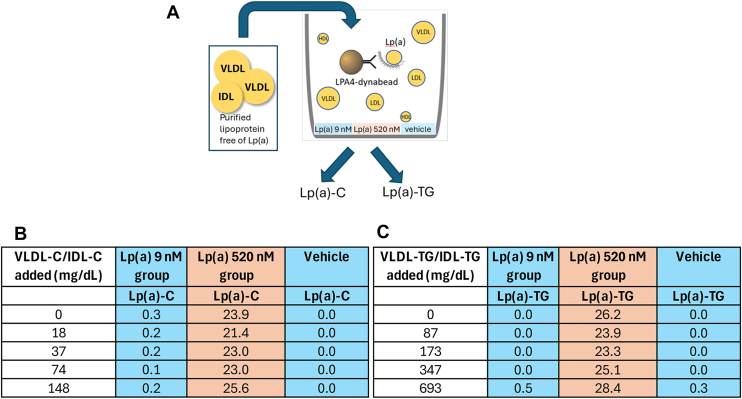
Assessment of interference from apo(a)-free VLDL/IDL in the Lp(a)-C and Lp(a)-TG assays. A: Experimental schematic illustrating spike-in of purified VLDL/IDL free of Lp(a) into plasma samples containing low (9 nmol/L) or high (520 nmol/L) Lp(a) concentrations, or vehicle control, followed by immune-isolation using LPA4-conjugated magnetic beads and enzymatic quantification of Lp(a)-C and Lp(a)-TG]. B: Lp(a)-C and (C) Lp(a)-TG measurements across increasing concentrations of added VLDL/IDL cholesterol or triglycerides demonstrate no measurable interference, confirming assay specificity for apo(a)-associated lipids. IDL, intermediate density lipoprotein; TG, triglyceride; Lp(a), lipoprotein(a); Lp(a)-TG, triglyceride associated with Lp(a); Lp(a)-C, Lp(a)-cholesterol.

VLDL/IDL was then spiked into plasma samples containing low (9 nmol/L) or high (520 nmol/L) Lp(a) concentrations across a wide range of TG and cholesterol concentrations. Addition of up to 693 mg/dl of VLDL/IDL TGs or 148 mg/dl of VLDL/IDL-C did not result in measurable increases in Lp(a)-TG or Lp(a)-C values (Fig. 2).

### Effect of washing buffers containing EACA and proline on Lp(a)-TG and Lp(a)-C measurements

To assess the contribution of nonspecific lipoprotein associations to measured Lp(a)-TG lipids, Lp(a)-TG and Lp(a)-C were quantified in 15 paired plasma samples from patients with varying TG levels (173–507 mg/dl) and with or without the addition of EACA and proline (Table 1). Inclusion of EACA and proline resulted in a consistent reduction in measured Lp(a)-TG across samples, with mean values decreasing from 19.0 ± 21.3 mg/dl to 14.7 ± 18.8 mg/dl, corresponding to a mean relative reduction of 29.7 ± 15.1%. Reductions in Lp(a)-TG were observed in all samples, ranging from 4.6% to 54.4%.

**Table 1:** Measurement of Lp(a)-TG and Lp(a)-C and without and with addition of EACA and proline

Patient	Triglycerides, mg/dl	Lp(a)-TG (mg/dl) Without EACA + Proline	Lp(a)-TG (mg/dl) With EACA + Proline	% Difference
1	323	76.1	60.0	−21.2
2	507	16.1	11.3	−29.9
3	387	20.0	19.1	−4.6
4	269	20.3	9.2	−54.4
5	414	13.5	10.6	−21.3
6	173	8.1	4.4	−45.5
7	311	7.7	6.3	−19.3
8	210	8.9	5.7	−36.1
9	213	12.8	8.9	−30.5
10	214	8.1	5.7	−29.8
11	202	5.9	2.8	−52.6
12	254	16.9	11.2	−34.0
13	263	4.1	2.4	−42.3
14	266	3.8	3.2	−18.0
15	452	62.9	59.4	−5.5
**Mean**		**19.0**	**14.7**	**−29.7**
**SD**		**21.3**	**18.8**	**15.1**

Patient	Lp(a), nmol/l	Lp(a)-C (mg/dl) Without EACA + Proline	Lp(a)-C (mg/dl) With EACA + Proline	% Difference

1	200	25.5	20.3	−20.2
2	19	2.3	2.5	9.6
3	11	7.9	6.8	−13.9
4	194	9.7	5.5	−42.7
5	21	3.3	3.0	−8.7
6	23	2.7	1.4	−46.8
7	16	2.4	1.7	−27.7
8	18	2.8	2.2	−23.4
9	176	8.9	5.5	−37.4
10	35	3.2	2.4	−25.4
11	191	7.5	5.2	−30.6
12	242	10.4	9.7	−7.1
13	15	1.6	1.0	−40.2
14	14	1.4	1.3	−5.9
15	219	17.4	15.9	−8.4
**Mean**		**7.1**	**5.6**	**−21.9**
**SD**		**6.8**	**5.7**	**16.1**

EACA, ε-aminocaproic acid; Lp(a), lipoprotein(a); Lp(a)-C, Lp(a)-cholesterol; Lp(a)-TG, triglyceride associated with Lp(a); TG, triglyceride.

Similarly, measured Lp(a)-C decreased following addition of EACA and proline, with mean values declining from 7.1 ± 6.8 mg/dl to 5.6 ± 5.7 mg/dl, corresponding to a mean relative reduction of 21.9 ± 16.1%. Individual changes in Lp(a)-C ranged from a 46.8% decrease to an increase of 9.6%.

These paired analyses demonstrate that inclusion of washing buffers substantially lowers measured Lp(a)-TG and Lp(a)-C signals, consistent with effective reduction of nonspecific interactions between apo(a)-containing particles and TG-rich lipoproteins.

### Lp(a)-TG TGs and cholesterol in normotriglyceridemic individuals

Baseline characteristics of the 36 normotriglyceridemic individuals are shown in Supplemental Table S1. The mean age was 64.6 ± 10.3 years, 56% were male, and the majority had angiographically documented coronary artery disease.

Mean fasting lipid levels were consistent with a normotriglyceridemic state, with plasma TG levels of 98.4 ± 31.9 mg/dl, total cholesterol of 151 ± 37 mg/dl, LDL-C of 80.9 ± 31.2 mg/dl, and HDL-C of 52.6 ± 17.4 mg/dl (Table 2). Mean Lp(a) concentration was 91.8 ± 101.5 nmol/L. Lp(a)-TG lipid content was low, with mean Lp(a)-C of 4.03 ± 4.01 mg/dl and Lp(a)-TG of 1.42 ± 2.83 mg/dl. Consistent with a predominantly cholesterol-rich, LDL-like Lp(a) particle composition, the mean Lp(a)-TG/Lp(a)-C ratio was low (0.59 ± 1.27), and Lp(a) lipid-to-particle molar ratios were correspondingly low.

**Table 2:** Laboratory variables in the normotriglyceridemic cohort and in patients with moderate hypertriglyceridemia

Variable	Normotriglyceridemic Cohort	Moderate Hypertriglyceridemia
Total cholesterol (mg/dl), mean (SD) mg/dL	151 (37)	156.7 (32.7)
Triglycerides (mg/dl), mean (SD)	98.4 (31.9)	284 (85)
Triglycerides (mg/dl), median (IQR)	97.5 (72–118)	261 (222–329)
LDL-C (mg/dl), mean (SD)	80.9 (31.2)	67.4 (27.2)
HDL-C (mg/dl), mean (SD)	52.6 (17.4)	34.8 (9.2)
Lp(a) (nmol/l), mean (SD)	91.8 (101.5)	66.1 (84.2)
Lp(a) (nmol/l), median (IQR)	101.5 (16.1–138.8)	23.8 (11.0–79.5)
Lp(a)-TG (mg/dl), mean (SD)	1.42 (2.83)	53.7 ± 25.3
Lp(a)-C (mg/dl), mean (SD)	4.03 (4.01)	14.4 ± 6.9
Lp(a)-TG/Lp(a)-C ratio	0.59 (1.27)	3.99 ± 1.2
Lp(a)-C/Lp(a) molar ratio	2,794 (4,066)	3,221 ± 3,052
Lp(a)-TG/Lp(a) molar ratio	1,291 (3,303)	1840 ± 1775

EACA, ε-aminocaproic acid; IQR, interquartile range; Lp(a), lipoprotein(a); Lp(a)-C, Lp(a)-cholesterol; Lp(a)-TG, triglyceride associated with Lp(a).

### Laboratory variables in hypertriglyceridemic individuals

Baseline laboratory characteristics of the 114 individuals with moderate hypertriglyceridemia are shown in Table 2. Mean fasting TGs were 284 ± 85 mg/dl, with elevated estimated VLDL-C (54.9 ± 17.5 mg/dl) and apolipoprotein C-III (16.2 ± 4.04 mg/dl), while mean total cholesterol, LDL-C by ultracentrifugation, and HDL-C were 156.7 ± 32.70 mg/dl, 67.4 ± 27.2 mg/dl, and 34.8 ± 9.2 mg/dl, respectively. Median (IQR) Lp(a) concentration was 23.8 nmol/L (11.0–79.5), with non-Lp(a) apoB comprising the majority of circulating apoB particles.

### Distribution of Lp(a)-TG/Lp(a)-C ratios

Lp(a)-TG lipid measures in individuals with moderate hypertriglyceridemia are summarized in Table 2. Mean Lp(a)-TG concentration was 53.7 ± 25.3 mg/dl, accounting for 18.9% of total plasma TGs. Mean Lp(a)-C concentration was 14.4 ± 6.9 mg/dl, representing 9.2% of total plasma cholesterol and ∼20% of LDL-C. The mean Lp(a)-TG/Lp(a)-C ratio was markedly elevated at 3.99 ± 1.2. Consistent with these findings, Lp(a)-TG and Lp(a)-C normalized to Lp(a) particle number were substantially higher, with mean Lp(a)-TG/Lp(a) and Lp(a)-C/Lp(a) ratios of 3,221 ± 3,052 and 1840 ± 1775, respectively.

### Correlations of Lp(a)-TG lipids with plasma lipoproteins

Spearman correlation analyses in individuals with moderate hypertriglyceridemia are shown in Table 3. Lp(a)-TG correlated strongly with plasma TGs (r = 0.59, *P* < 0.0001), apolipoprotein C-III (r = 0.43, *P* < 0.0001), and estimated VLDL-C (r = 0.53, *P* < 0.0001), and inversely with HDL-C (r = −0.42, *P* < 0.0001) and apolipoprotein A-I (r = −0.28, *P* = 0.0076). Lp(a)-C showed similar positive correlations with plasma TGs (r = 0.58, *P* < 0.0001), apolipoprotein C-III (r = 0.40, *P* = 0.0001), estimated VLDL-C (r = 0.57, *P* < 0.0001), and non–HDL-C (r = 0.33, *P* = 0.0015), and an inverse correlation with HDL-C (r = −0.29, *P* = 0.0051).

**Table 3:** Spearman correlations of key lipoprotein variables in patients with moderate hypertriglyceridemia

Analyte in Lipoprotein Data	Lp(a)-TG		Lp(a)-C		Lp(a)	
Statistics	r^a^	*P* value	r^a^	*P* value	r^a^	*P* value
Baseline						
Apolipoprotein C-III	0.43	<0.0001	0.40	0.0001	−0.16	0.09
Triglycerides	0.59	<0.0001	0.58	<0.0001	−0.11	0.25
Total cholesterol	−0.05	0.66	0.25	0.018	0.15	0.11
Estimated VLDL-C	0.53	<0.0001	0.57	<0.0001	−0.05	0.61
Apolipoprotein B	0.01	0.91	0.24	0.022	0.26	0.0062
LDL-C by ultracentrifugation	−0.13	0.21	0.09	0.43	0.20	0.039
HDL-C	−0.42	<0.0001	−0.29	0.005	0.04	0.66
Non-HDL-C	0.07	0.52	0.33	0.0015	0.15	0.11
Apolipoprotein A1	−0.28	0.0076	−0.16	0.14	−0.01	0.87
Lp(a)-ApoB	0.23	0.029	0.43	<0.0001	1.00	<0.0001
non–Lp(a)-ApoB	−0.02	0.87	0.14	0.20	0.04	0.67
Lp(a)	0.23	0.029	0.41	<0.0001	-	-

EACA, ε-aminocaproic acid; Lp(a), lipoprotein(a); Lp(a)-C, Lp(a)-cholesterol; Lp(a)-TG, triglyceride associated with Lp(a).

a Spearman correlation coefficient.

In contrast, Lp(a) particle concentration showed weaker or nonsignificant correlations with TRL measures but correlated modestly with apolipoprotein B (r = 0.26, *P* = 0.0062) and LDL-C by ultracentrifugation (r = 0.20, *P* = 0.0385).

### Contribution of Lp(a)-associated lipids to plasma tTG, total cholesterol, and LDL-C pools

In normotriglyceridemic individuals, low absolute concentrations of apo(a)-associated lipids were matched by correspondingly small fractional contributions to circulating lipid pools. Mean Lp(a)-TG concentration of 1.42 ± 2.83 mg/dl accounted for mean (SD) 1.22% (0.10) of total plasma TGs, while mean [Lp(a)-C concentration of 4.03 ± 4.01 mg/dl represented 2.62% (2.01) of total plasma cholesterol.

In contrast, in individuals with moderate hypertriglyceridemia, higher absolute concentrations of apo(a)-associated lipids were closely matched by proportionally larger contributions to plasma lipid pools. Mean Lp(a)-TG concentration of 53.7 ± 25.3 mg/dl corresponded to mean (SD) 19.9% (6.53) of total fasting plasma TGs. Mean Lp(a)-C concentration of 14.4 ± 6.9 mg/dl accounted for 9.68% (4.41) of total plasma cholesterol and nearly 21% of measured LDL-C by ultracentrifugation.

## Discussion

In this study, we developed and validated a novel immune-isolation assay enabling direct quantification of TGs associated with apo(a)-containing particles [Lp(a)-TG]. Using this approach, we demonstrate that apo(a)-associated TGs are low in normotriglyceridemic individuals but markedly higher in individuals with moderate hypertriglyceridemia. These findings provide quantitative evidence that the lipid composition of Lp(a) is variable and influenced by the presence of hypertriglyceridemia.

Our findings extend prior observations regarding Lp(a) heterogeneity by providing quantitative evidence that TG enrichment of Lp(a) is substantial in hypertriglyceridemic states. Compositional studies of Lp(a) isolated by density ultracentrifugation demonstrated that the mass of Lp(a) from healthy volunteers was composed of 25%–30% protein, 30%–38% cholesteryl esters, 4%–6% free cholesterol, 2%–9% TGs, 15%–19% phospholipids, and 8%–15% carbohydrate of a total mass of the intact Lp(a) particle of 3.400,000–3.800,000 Da (12). However, several postprandial and hypertriglyceridemic studies have suggested that apo(a) can associate with TRLs, including chylomicron remnants, VLDL, and IDL particles (16, 18, 27, 28, 29). Western blot and immunochemical analyses demonstrated apo(a) immunoreactivity within TRL density fractions, particularly in individuals with impaired lipolysis or elevated apolipoprotein C-III levels.

In this study, consistent with prior compositional studies in normolipidemic individuals, TGs comprise a small fraction of Lp(a) lipid mass under normal metabolic conditions. However, the marked elevation in the Lp(a)-TG/Lp(a)-C ratio in the setting of moderate hypertriglyceridemia is consistent with apo(a) being associated with larger, TG-enriched particles that resemble remnant-like lipoproteins, rather than the cholesterol-rich, LDL-like Lp(a) typically observed under normolipidemic conditions. Importantly, the present data should be interpreted as reflecting apo(a)-associated TGs, rather than structural incorporation of TGs into a stable Lp(a) particle. The correlations of Lp(a)-TG with plasma TGs, apolipoprotein C-III, and estimated VLDL-C, coupled with weaker associations with Lp(a) particle concentration itself, suggest that TG enrichment of Lp(a) is driven primarily by the metabolic environment rather than by intrinsic differences in Lp(a) synthesis. The present data should be interpreted as reflecting TGs associated with apo(a)-associated particles rather than definitive incorporation into a stable Lp(a) core. Such enrichment may reflect dynamic lipid exchange in hypertriglyceridemic states, potentially influenced by prolonged residence time of TRLs or lipid transfer protein–mediated pathways, although these mechanisms were not directly assessed.

The observation that Lp(a)-TG accounted for approximately 20% of total plasma TGs in individuals with moderate hypertriglyceridemia is notable and concordant with the marked elevation in absolute Lp(a)-TG concentrations observed in this metabolic state. In contrast, in normotriglyceridemic individuals, the very low absolute Lp(a)-TG concentrations were matched by only a negligible contribution to the total plasma TG pool. A similar pattern was observed for Lp(a)-C, which accounted for approximately 10% of total plasma cholesterol and nearly 20% of measured LDL-C in hypertriglyceridemic individuals, whereas its contribution was minimal under normotriglyceridemic conditions. Together, these findings demonstrate that the fractional contribution of apo(a)-associated lipids to circulating TG and cholesterol pools closely tracks the underlying metabolic environment rather than Lp(a) particle concentration alone. Although the proportional contribution of Lp(a)-C observed in hypertriglyceridemia is higher than that typically reported in individuals with low Lp(a) levels, it is comparable to values described in patients with markedly elevated Lp(a) in range of 200–250 nmo/l (22, 23, 26, 30), underscoring the potential for substantial Lp(a)-associated lipid burden even at modest Lp(a) particle concentrations when TG metabolism is perturbed. Although it is conceivable LDL and HDL particles are also enriched in TGs in hypertriglyceridemic states, the present study was not designed to determine whether TG enrichment of Lp(a) is universal or distinct relative to other lipoprotein classes, nor whether any quantitative or mechanistic differences would carry unique functional or atherogenic implications.

Although this study was not designed to assess clinical outcomes or TG subspecies, TG enrichment of apo(a)-containing particles may have functional relevance, analogous to emerging evidence linking elevated LDL TG content to atherosclerotic risk (31). In recent work, Dzobo *et al.* (32) demonstrated that diacylglycerols are enriched on Lp(a) particles and can directly promote monocyte inflammation through NF-κB and NLRP3 inflammasome–dependent pathways, providing functional evidence that noncholesterol lipids on Lp(a) may contribute to its proinflammatory properties. Together with these findings, the present ability to quantify Lp(a)-associated TGs highlights the broader heterogeneity of the Lp(a) lipidome and provides a platform for future studies to determine whether specific neutral lipid species or metabolic remodeling of Lp(a) influence vascular inflammation and atherogenic risk.

The clinical relevance of these observations of higher TG and cholesterol content of Lp(a) in patients with moderate hypertriglyceridemia requires further study. It was recently demonstrated that elevated concentration of LDL TGs, measured with a direct assay, was associated with an increased risk of atherosclerotic cardiovascular disease (31). The ability to measure both Lp(a)-TG and Lp(a)-C with sensitive and high-throughput measurements will allow testing of whether these variables provide independent information on pathophysiology and cardiovascular risk prediction relative to established biomarkers.

This study has several limitations. The cross-sectional design and reliance on fasting samples preclude assessment of postprandial dynamics, where apo(a)-TRL interactions may be most pronounced. Apo(a) isoform size was not directly examined, and its influence on lipid association warrants future investigation. In addition, the roles of lipid transfer proteins and hepatic lipase activity were not evaluated.

In conclusion, we describe the first validated, high-throughput method for direct quantification of TGs associated with apo(a). Our findings demonstrate that apo(a)-associated lipid content is highly variable and strongly influenced by TG metabolism. This assay provides a foundation for future mechanistic, epidemiologic, and interventional studies aimed at defining how metabolic states and treatments that affect Lp(a) and TGs modify Lp(a) composition and function.

## Data Availability

Data that support the plots within this publication and other findings of this study are available from the corresponding authors upon request.

## Supplemental Data

This article contains supplemental data.

## Conflict of interest

S. T. is a coinventor and receives royalties from patents owned by University of California San Diego (UCSD), is a cofounder and has an equity interest in Oxitope, LLC, Kleanthi Diagnostics, LLC and Megaron, Inc., and has a dual appointment at UCSD and Ionis Pharmaceuticals. The other authors declare that they have no conflicts of interest with the contents of this article.
