# Supplementary material for: A novel immune-isolation method for direct quantification of triglycerides associated with lipoprotein(a)
Source: J Lipid Res. 2026 Feb 10;67(3):100996. doi: 10.1016/j.jlr.2026.100996 (PMC12993128; doi:10.1016/j.jlr.2026.100996)
Supplement: Supplemental Tables S1 and S2 [file mmc1.docx]

**Table S1.** Demographic and laboratory variables in 36 normotriglyceridemic (<175 mg/dL) subjects.

| **Variable** | **Value** |
| --- | --- |
| Age, mean (SD) | 64.6 (10.3) |
| Male sex, N (%) | 20 (56) |
| Race |  |
| White, N (%) | 24 (67) |
| Asian, N (%) | 4 (11) |
| Black, N (%) | 1 (3) |
| Hispanic, N (%) | 6 (17) |
| Other, N (%) | 1 (3) |
| CAD Status |  |
| No angiographic CAD, N (%) | 3 (8) |
| Non-obstructive CAD, N (%) | 5 (13.9) |
| Obstructive CAD (>70%), N (%) | 24 (66.7) |
| Acute MI Presentation, N (%) | 7 (19.4) |
| PCI, N (%) | 19 (52.7) |
| CABG, N (%) | 4 (11.1) |
| Co-morbidities |  |
| DM, N (%) | 8 (22.2) |
| HTN, N (%) | 21 (58.3) |
| Tobacco, N (%) | 4 (11.1) |

CAD subcategories sum to >100% due to overlapping categories (MI, PCI, CABG).

**Table S2.** Spearman correlations of key lipoprotein variables in patients with moderate hypertriglyceridemia.

|  | | TG | | LDL-C | | HDL-C | | Lp(a) | | Lp(a)-C | | Lp(a)-TG | |  |
| --- | --- | --- | --- | --- | --- | --- | --- | --- | --- | --- | --- | --- | --- | --- |
| TC | Correlation Coefficient | | 0.099 | | **0.843** | | **.629** | | 0.218 | | **0.411** | | -0.167 | |
|  | Sig. (2-tailed) | | 0.564 | | **<0.001** | | **<0.001** | | 0.202 | | **0.013** | | 0.331 | |
| TG | Correlation Coefficient | |  | | 0.162 | | **-0.374** | | -0.072 | | 0.071 | | **0.330** | |
|  | Sig. (2-tailed) | |  | | 0.367 | | **0.032** | | 0.677 | | 0.679 | | **0.049** | |
| LDL-C | Correlation Coefficient | |  | |  | | 0.211 | | 0.261 | | **0.451** | | 0.004 | |
|  | Sig. (2-tailed) | |  | |  | | 0.239 | | 0.142 | | **0.008** | | 0.984 | |
| HDL-C | Correlation Coefficient | |  | |  | |  | | 0.070 | | 0.188 | | -0.326 | |
|  | Sig. (2-tailed) | |  | |  | |  | | 0.698 | | 0.294 | | 0.064 | |
| Lp(a) | Correlation Coefficient | |  | |  | |  | |  | | **0.829** | | -0.137 | |
|  | Sig. (2-tailed) | |  | |  | |  | |  | | **<0.001** | | 0.426 | |
| Lp(a)-C | Correlation Coefficient | |  | |  | |  | |  | |  | | 0.045 | |
|  | Sig. (2-tailed) | |  | |  | |  | |  | |  | | 0.795 | |
